# Use of direct-acting anticoagulants (DOACs) delays surgery and is associated with increased mortality in hip fracture patients

**DOI:** 10.1007/s00068-024-02532-9

**Published:** 2024-05-07

**Authors:** Mads Sundet, Ulf Sundin, Aasmund Godø, Kristian Sydnes, Haldor Valland, Joseph Sexton, Mette Martinsen, Maren Paus, Hans Schmidt Rasmussen, Siri Lillegraven

**Affiliations:** 1 Department of Orthopedic Surgery, Diakonhjemmet Hospital, Vinderen, Box 23, N-0319 Oslo, Norway; 2 Department of Anesthesia, Diakonhjemmet Hospital, Vinderen, Box 23, N-0319 Oslo, Norway; 3 Center for Treatment of Rheumatic and Musculoskeletal Diseases (REMEDY), Diakonhjemmet Hospital, Oslo, Norway; 4 Faculty of Medicine, University of Oslo, Oslo, Norway

**Keywords:** Hip fracture, Mortality, Anesthesia, Surgical delay, Direct-acting oral anticoagulants

## Abstract

**Purpose:**

Treatment with direct-acting oral anticoagulants (DOACs) is increasing among hip-fracture patients, with accompanying safety concerns regarding spinal anesthesia (SA). The aim of this study was to investigate if DOAC use is associated with increased waiting time before surgery, increased mortality, or other adverse events.

**Methods:**

Registry data on surgically treated hip-fracture cases at a single hospital between 2015 and 2021 were analyzed. Multivariable regression analyses were performed with DOAC-status and choice of anesthesia as exposures, and waiting time, length of stay, transfusion, and mortality as outcomes.

**Results:**

2885 cases were included, 467 patients (16%) were using DOACs. DOAC users were older (86.3 vs. 82.2 years, p < 0.001), had a higher Charlson Comorbidity Index (2.1 vs. 1.5, p < 0.001) and had longer median time to surgery than non-DOAC cases (36 h vs 17 h, p < 0.001). General anesthesia (GA) was used in 19.3% of DOAC patients and in 3.0% of non-DOAC patients. DOAC-patients had an increased risk of one-month mortality (Adjusted Odds Ratio (AOR) 1.6 (1.1–2.3)) and one-year mortality (AOR 1.4 (1.1–1.8)). There were no differences in risk of blood transfusion. Patients on DOAC operated under GA had a lower risk of one-year mortality (AOR 0.5 (0.3–0.9)), but a similar one-month mortality to DOAC-patients operated under SA.

**Conclusion:**

DOAC users had a longer waiting time to surgery, indicating postponement of surgery due to concerns of the safety of SA. The clinical practice should be changed to allow earlier surgery for DOAC patients.

**Supplementary Information:**

The online version contains supplementary material available at 10.1007/s00068-024-02532-9.

## Introduction

Hip fractures in the elderly are associated with high mortality and morbidity, and optimizing the medical and surgical treatment of these patients is important. Norway has one of the highest incidences of hip fractures in the world [1], and the timely treatment of these fractures is often a logistical challenge. According to national Norwegian treatment recommendations, hip fractures should preferably be operated within 24 h, or at least within 48 h. NICE guidelines for the treatment of hip fractures state that surgery should be performed on the day of admission, or the day after [2]. Although there is conflicting evidence about the safety of waiting up to 48 h after admission, it is well established that waiting more than 48 h is associated with increased mortality and complications [3–5].

Direct-acting oral anticoagulants (DOACs) are a group of drugs inhibiting factor Xa (rivaroxaban, apixaban and edoxaban), and one direct thrombin inhibitor (dabigatran). DOACs were introduced in the early 2000s and are widely used in patients with atrial fibrillation, and in patients with a high risk of venous thromboembolism. DOACs inhibit coagulation, and there is a concern that spinal anesthesia in patients on DOACs may be associated with an increased risk of spinal hematomas. Because of this, delaying spinal anesthesia for 24–72 h after intake of DOACs is recommended in several current guidelines [6, 7], with the length of the delay depending of type of drug, renal function and dosage.

There is a concern that DOAC use may lead to delayed hip fracture treatment. This has been studied in some cohorts. One study of 3455 hip fracture patients found increased waiting time for closed reduction and internal fixation for DOAC patients, but not for hemiarthroplasty [8]. In a study from the United States, 41 of 535 hip fracture patients used DOACs, and there were no differences in waiting time before surgery and no differences in complications and mortality between the groups [9]. In a study from Bergen, Norway, 47 DOAC users among 314 hip fractures had no significant differences in waiting time and risk of complications and death [10]. A large register study from the Danish Multidisciplinary Hip Fracture Registry found increased surgical delay and a slightly increased risk of blood transfusion in DOAC users, but no increase in 30-day mortality [11]. In a review article on the subject, the authors identified 21 papers and found that surgical delay was reported in most papers, but there was no clear evidence of increased mortality, blood loss, transfusion rates, complication rates, re-operations or readmissions in DOAC users [12].

The aim of this study was to investigate if surgery is delayed in DOAC users, and if there are associations between DOAC use and complications and mortality. We also wanted to investigate whether spinal anesthesia (SA) or general anesthesia (GA) was associated with the rate of mortality in DOAC patients.

## Materials and methods

### Treatment setting

Diakonhjemmet Hospital treats 450 patients with hip fractures yearly, within a dedicated orthogeriatric unit. Hip fractures are operated during daytime and the evening, but not during the night (23–08). Most procedures are done by orthopedic surgeons in training, often assisted by consultant orthopedic surgeons. There are no guidelines restricting the timing of the surgery itself after intake of DOACs. Anesthesia is provided by the anesthesiologist on call, and the choice of anesthesia is based on this anesthesiologist’s clinical judgment. In our department, SA is the preferred mode of anesthesia in hip fracture patients. This is because SA gives good postoperative pain relief and because GA is viewed as more complicated in patients with respiratory and cardiovascular comorbidities. Internal guidelines state that SA should be delayed 48 h after intake of DOACs. Patients who have been using DOACs can be operated in GA at any time, if the anesthesiologist considers this to be safe.

### The database

Since 2006, all patients with hip fractures have been registered in an internal registry (Diakonhjemmet Hip Fracture Registry (DHFR)). The database contains data about comorbidities, medication, treatment, complications, demographic information, laboratory tests, fracture classification, operation type, ASA class, and time of surgery. The information is collected from the electronic patient file by a trained nurse after discharge of the patient, and information about deaths are collected from the Norwegian National Population register.

Information about DOAC use has been systematically recorded from 2015, and patients treated before this time were excluded from the analysis. To ensure complete one-year mortality data, patients treated after 31.12.2021 were also excluded.

**Fig. 1:**
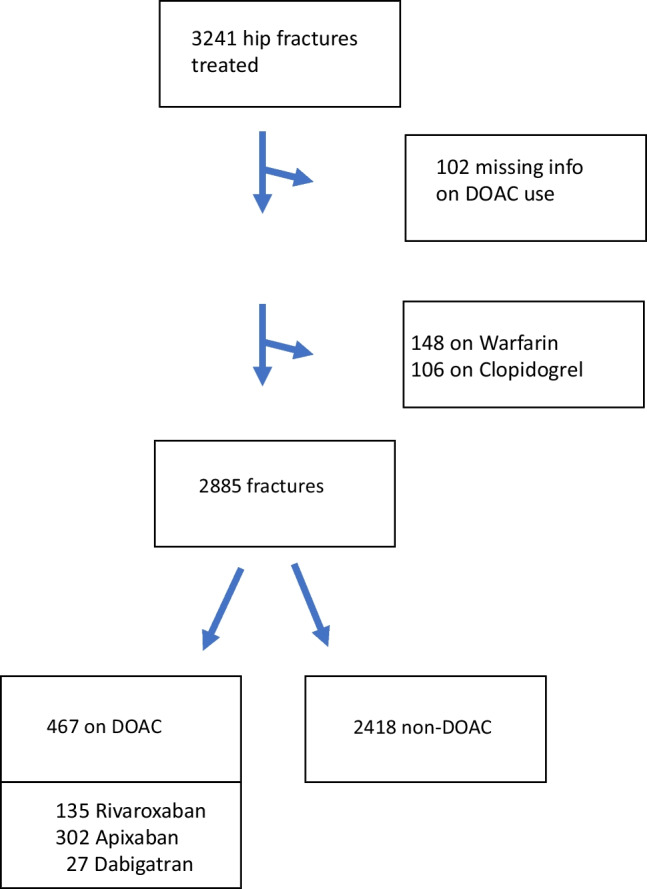
Flow chart of the patients included in the study

Warfarin and clopidogrel are anticoagulants that are not DOACs, but patients using these drugs have related comorbidities and the same considerations concerning spinal anesthesia, and these patients were therefore excluded from the study. Patients using acetylsalicylic acid and/or dipyridamole were included in the non-DOAC group. Figure 1 contains a flowchart of study subject inclusion.

### Exposures and outcomes

The main exposure in this study was the use of DOACs (rivaroxaban, apixaban, edoxaban or dabigatran). We also assessed the choice of anesthesia (SA or GA) in DOAC patients. We had no information about the timing of the last intake of DOACs.

The main outcome in this study was waiting time between admission and surgery, reported in hours. Other outcomes were blood transfusion (yes/no), length of stay (days), death within a month (30 days) after admission, and death within a year (365 days) after admission.

### Data analysis

**Fig. 2:**
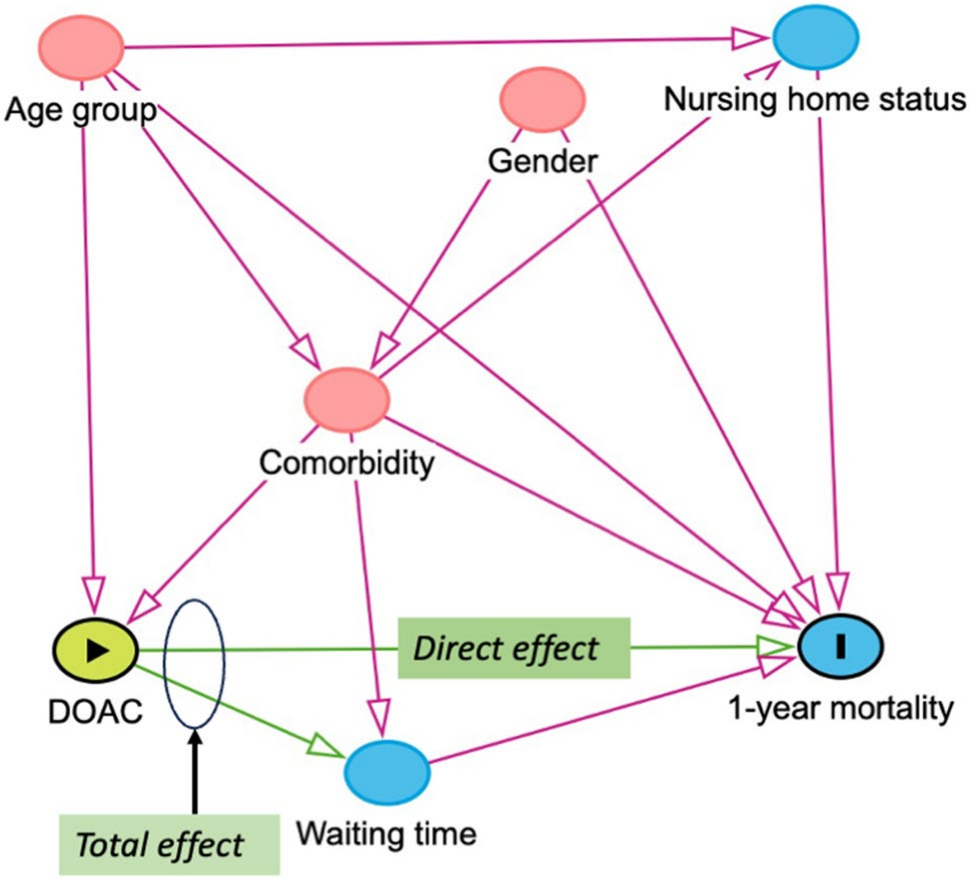
Directed acyclic graph of the association between DOAC use and survival. In this analysis the red covariates are confounders, and should by adjusted for. Waiting time is a mediator between DOAC-use and mortality, and should not be adjusted for to measure the total effect. When investigating the direct effect of DOACs on mortality, waiting time should be adjusted for (source: dagitty.net)

Each hip fracture was analyzed as a separate case, although some patients had a repeated admission for a contralateral fracture. Demographic factors were described by means or frequencies (percent), grouped by whether they used DOACs or not. The statistical significance of differences in proportions was tested with chi-square tests, differences in means were tested with linear regression, and differences in medians were tested with quantile regression. The outcomes *waiting time* and *hospital stay* were right-skewed and not normally distributed, but due to the large number of observations the assumptions for linear regression were met. Directed acyclic graphs (DAGs) were utilized to develop a model of the causal relationship between DOAC use as an exposure, and mortality as an outcome (Fig. 2). The Charlson Comorbidity Index (CCI), age, and gender were chosen as adjustment covariates when analyzing all outcomes. Nursing home status (yes/no) was also included as an adjustment factor in the analysis of outcomes of SA or GA in DOAC patients. Waiting time before surgery was viewed as a mediator on the causal paths between DOAC use/choice of anesthesia and mortality (Fig. 2) and was not adjusted for in the analysis. To analyze the *direct effect* of DOAC use on mortality, waiting time grouped into categorical categories were included as an adjustment covariate. Directed Acyclic Graphs for the other analyses, made with Dagitty software [13], are available in the online supplementary information. The effect of age was non-linear, and age was therefore analyzed as a categorical covariate by decades. The CCI had a linear association with mortality and was used as a continuous covariate. There were 2% missing values for nursing home status, and these were excluded in the one analysis where this was used for adjustment. There were no missing values for the other adjustment covariates, and no imputation was necessary. All analyses were done using STATA version 17.0 (StataCorp, College Station, Tx, USA).

### Ethical issues

The study has been performed in accordance with the standards of the Helsinki declaration. The database is approved by our hospital research board and our data protection officer. Patients are informed about their registrations in the database, but do not provide a formal written consent. This is in accordance with Norwegian legislation about research on data from quality databases. No harm is conceivable for the patients by inclusion in the study.

## Results

During the years 2015–2021, 3241 hip fractures were treated. Of these, 2885 hip fractures in 2675 patients were included in the analysis (Fig. 1), 2074 of them females (72%). We found that that DOAC use was increasing with increased age and comorbidity, as shown in Table 1, where demographical and clinical data on the patients are reported. DOAC-users waited a median of 36 h (95% CI 35–38), and stayed in the hospital for a median of 6 days (95% CI 6–6), while non-users waited a median of 17 h (95% CI 17–18) and stayed in the hospital for a median of 7 days (95% CI 7–7). The differences were statistically significant (p < 0.001). A multivariable linear regression analysis confirmed these differences (Table 2). There were no differences in transfusions between DOAC users and non-users, but DOAC users had an increased risk of waiting more than 24 h or 48 h before surgery, and increased one-month, and one-year mortality rates (Table 3). Waiting time was viewed as a mediator between DOAC-use and mortality, and when this was included in the model to calculate the *direct effect* of DOAC use on mortality (Fig. 2), the effect estimate decreased and was no longer statistically significant.

**Table 1:** Demographics and comorbidities

	Non-DOAC	DOAC	*p*-value
Total (%)	2418 (83.8)	467 (16.2)	
Female gender	72.7%	67.7%	0.03
Mean age (SD)	82.2 (10.6)	86.3 (7.8)	< 0.001
Patients with dementia	29.1%	28.9%	0.97
Nursing home residents	23.0%	22.8%	0.90
Age group (%)			
< 60	75 (3.1)	4 (0.9)	
60–70	262 (10.8)	14 (3.0)	
70–80	595 (24.6)	84 (18.0)	
80–90	933 (38.6)	205 (43.9)	
90 +	553 (22.9)	160 (34.2)	
Mean Charlson Comorbidity Index (95% CI)	1.5 (1.4–1.6)	2.1 (1.9–2.2)	< 0.001
Charlson comorbidity index (%)			
0	770 (31.8)	72 (15.4)	
1	713 (29.5)	128 (27.4)	
2	456 (18.9)	110 (23.6)	
3–4	355 (14.7)	118 (25.3)	
5–6	79 (3.3)	27 (5.8)	
7 +	45 (1.7)	12 (2.6)

**Table 2:** Linear regression analysis of continuous outcomes

	Non-DOAC	DOAC	Difference (95% CI)	Adjusted difference (95%CI)*	*p*
Waiting time for surgery, mean ((95% CI)	18.3 (17.9 -18.8)	34.3 (33.0–35.6)	**15.9 (14.8–17.1)**	**16.0 (14.8–17.1)**	** < 0.001**
Length of stay, mean (95% CI)	5.9 (5.8–6.0)	6.9 (6.6–7.1)	**1.0 (0.7–1.3)**	**1.0 (0.8–1.4)**	** < 0.001**

*Adjusted for gender, age groups and CCI

**Table 3:** Binary outcomes: results from logistic regression

	Non-DOAC	DOAC	Crude OR^ab^	Adjusted OR^abc^	Adjusted OR: Direct effect ^abd^
General anesthesia	3.0%	19.3%	**7.7 (5.5–10.9)**	**9.6 (6.6–13.9)**	
Transfusion	21.9%	26.6%	**1.3 (1.03–1.6)**	1.0 (0.8–1.3)	
Waiting time more than 24 h	26.1%	73.7%	**7.9 (6.3–9.9)**	**7.9 (6.3–10.0)**	
Waiting time more than 48 h	2.0%	13.5%	**7.7 (5.2–11.3)**	**7.8 (5.2–11.7)**	
One month mortality	5.6%	12.0%	**2.3 (1.6–3.2)**	**1.60 (1.14–2.27)**	1.46 (0.99–2.15)
One year mortality	20.7%	33.4%	**1.9 (1.5–2.4)**	**1.34 (1.06–1.70)**	1.17 (0.90–1.52)

^a^OR: Odds Ratio

^b^Non-DOAC set as reference value, bold font indicates statistical significance

^c^Adjusted for gender, age groups and CCI

^d^Adjusted for gender, age groups, CCI and waiting time before surgery

### Anesthesia

Among non-DOAC users, 3% (60/1995) received GA, while the corresponding percentage for DOAC users was 19% (88/456) (p < 0.001). In DOAC users, the one-month mortality rate was similar between the types of anesthesia, but the one-year mortality rate was lower in the GA group (Table 4). DOAC-patients treated with GA had a mean waiting time from admission to surgery of 21.6 h while those treated with SA waited 37.2 h in average (p < 0.001).

**Table 4:** Anesthesia method and mortality in DOAC-patients, analyzed by logistic regression

	Spinal anesthesia	General anesthesia	Crude OR^ab^	Adjusted OR ^abc^
One month mortality	44/368 patients (12.0%)	10/88 patients (11.4%)	0.9 (0.5–2.0)	1.1 (0.4–3.2)
One year mortality	130/368 patients (35.3%)	19/88 patients (21.5%)	**0.5 (0.3–0.9)**	**0.5 (0.3–0.9)**

^a^OR: Odds Ratio

^b^Spinal anesthesia set as reference category

^c^Adjusted for gender, nursing home status, age groups and CCI

### Mortality, DOAC use, and waiting time

**Fig. 3:**
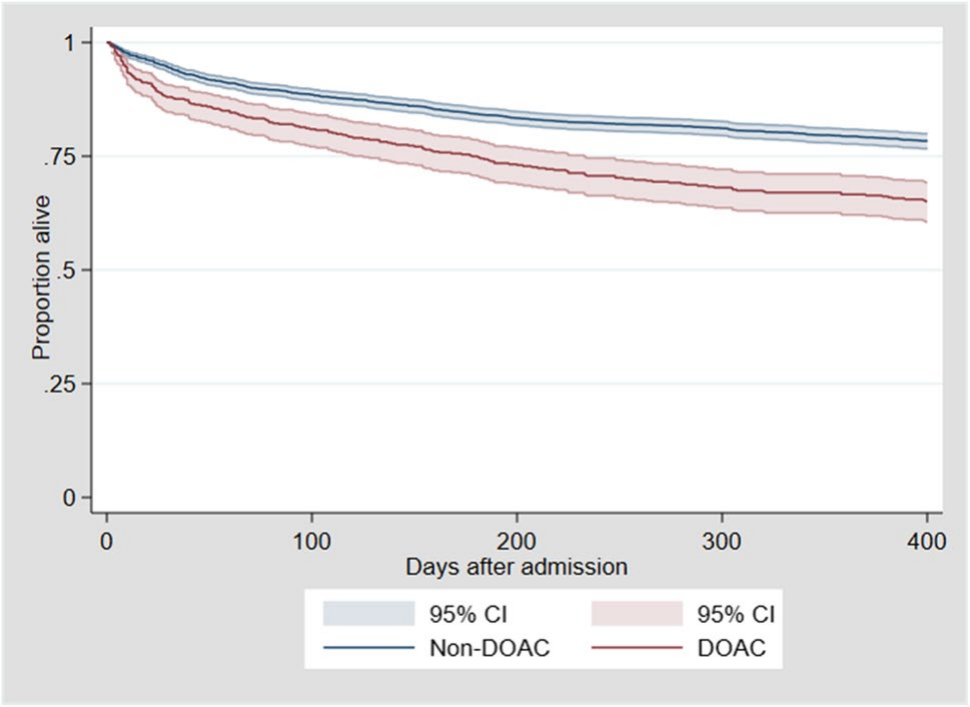
Survival after admission for a hip fracture, subgraphs for DOAC-users and non-users

Figure 3 illustrates the increased mortality in DOAC-patients during the first 400 days after admission. Analysis of the *direct effect* of DOACs (the effect not going through the mediator *waiting time* in Fig. 1), is done by additionally adjusting for waiting time, and this analysis is seen in Table 3. When this is included in the model, the effect of DOAC use decreases for the one-month mortality, and more profoundly for the one-year mortality, and both findings lose their statistical significance, showing that at least part of the effect of DOAC use on mortality is mediated through the increased waiting time before surgery.

## Discussion

In our study, patients who use DOAC have a more than twice as long median waiting time from admission to surgery for hip fractures than non-DOAC patients. We also demonstrated that patients using DOACs have a higher 30-day mortality and one-year mortality rate. Patients on DOAC operated under general anesthesia had a much shorter waiting time than patients on DOAC operated with spinal anesthesia.

Earlier studies show conflicting results about the increase in surgical delay for DOAC-patients. This is not surprising, as this is highly dependent on the local practice at each hospital. Some hospitals focus on limiting the delay, either by earlier spinal anesthesia, or by choosing general anesthesia more often. Is it important to note that the guidelines concerning DOACs and the timing of spinal anesthesia are not uniform. To give an example, the guidelines published by the Association of Anaesthetists of Great Britain & Ireland allows spinal anesthesia in hip fracture to be performed 24 h after intake of rivaroxaban, apixaban and edoxaban, except in cases with renal failure [14]. This is in contrast to our local guidelines, that has mandated a 48-h delay, regardless of renal function. The most influential guidelines on the subject does not differentiate between elective and emergency surgery [6, 7].

The increased mortality rate in DOAC patients is perhaps not surprising, given that DOAC-use is an indication of the presence of cardiovascular or thromboembolic disease, which are frequent causes of death. This has however not been shown in other studies, except in the one by Schermann et al. [8], where the DOAC-users that underwent closed reduction and internal fixation of their hip fractures had higher mortality. When adjusting for waiting time before surgery, the association between DOACs and one-year mortality became weaker and not statistically significant, suggesting that part of the negative effects of DOAC use on mortality is mediated through increasing the waiting time. This effect was strongest when analyzing one-year mortality, and less strong for one-month mortality, which is paradoxical; one should think that the effect of waiting long before surgery should be more visible in short term outcomes than in long term outcomes. The same applies to the finding that DOAC-patients operated under spinal anesthesia had a similar one-month mortality as patients operated under general anesthesia, but an increased one-year mortality. This suggests that there is unmeasured confounding involved that is associated with both one-year mortality, DOAC use and the choice of spinal anesthesia: for example, cardiopulmonary disease that is not sufficiently adjusted for when adjusting for the Charlson Comorbidity Index. It is worth noting that atrial fibrillation or previous venous thrombosis, which are common indications for DOACs, are not among the diseases that increases the Charlson Comorbidity Index. This means that the findings regarding mortality and the choice of anesthesia should be interpreted with caution. The registry also did not contain information about events occurring after discharge from hospital, and we were not able to assess whether there were differences in complications and reoperations between DOAC-users and non-users that could explain the increased mortality.

These data warrant a change of practice in the treatment of hip fracture patients using DOACs. One approach would be to increase the use of GA in DOAC-patients, a strategy that seems to have yielded good results in another Norwegian study where they found no increase in surgical delay [10]. In this study they administered GA in 47% of the DOAC-patients, in contrast to 19% in our patients. Another viable strategy is to administer SA earlier after intake of DOACs, which is viewed as acceptable in the Great Britain and Ireland guidelines for anesthesia in hip fracture patients [14].

The results reported in this study reflect the local practice of our hospital, where there is a strong preference for spinal anesthesia and a strict enforcement of a 48-h delay of spinal anesthesia after intake of DOACs. This might limit the generalizability to other settings, and we believe that there are large variations between different countries and between hospitals within the same country.

One of the limitations in our study is that we had no information about the time of the last intake of DOACs. It was thus not possible to find the time between intake and anaesthesia, as DOACs are taken at different times by different patients and patients frequently report missing their doses. Some patients on DOACs were operated within 24 h after *admission* with spinal, but our interpretation of this (based on our clinical experience from the hospital) is that these patients still had not been taking DOACs the last 48 h (e.g. because they forgot to take their medication the day before, because of delay in hospitalisation, or because they were unable to call for help after fracture).

Other limitations include that for some analyses, such as the choice of anesthesia in DOAC users, some of the subgroups are still small. The database also has very limited information about adverse events and complications, such as infections and reoperations. The main strength of this study is the large clinical cohort on DOAC use in hip fractures. The data collection was standardized and prospective, and it had a small number of missing values.

## Conclusion/interpretation

In conclusion, we have demonstrated that the practice of preferring spinal anesthesia for hip fracture patients and at the same time delaying this anesthesia until 48 h after intake of DOAC leads to a delay of surgical treatment in DOAC patients. DOAC patients had higher mortality, both after one month and one year. The practice of delaying spinal anesthesia after intake of DOACs varies across regions and hospitals, and there is a need to develop and implement new international guidelines for the anesthesia of hip fracture patients to address this problem. Further research, including randomized prospective trials, is warranted to identify the optimal treatment strategies for these patients.

## Supplementary Information

Below is the link to the electronic supplementary material.

Supplementary file1 (DOCX 9775 KB)

## Data Availability

No datasets were generated or analysed during the current study.
