# Supplementary material for: Use of direct-acting anticoagulants (DOACs) delays surgery and is associated with increased mortality in hip fracture patients
Source: Eur J Trauma Emerg Surg. 2024 May 7;50(4):1851–7. doi: 10.1007/s00068-024-02532-9 (PMC11458687; doi:10.1007/s00068-024-02532-9)

# Appendix

Appendix figure 1: Directed Acyclic Graph for the adjustment model for the association between the choice of anesthesia and mortality in DOAC patients. Waiting time is a mediator between the choice of anesthesia and mortality, and should thus not be adjusted for.


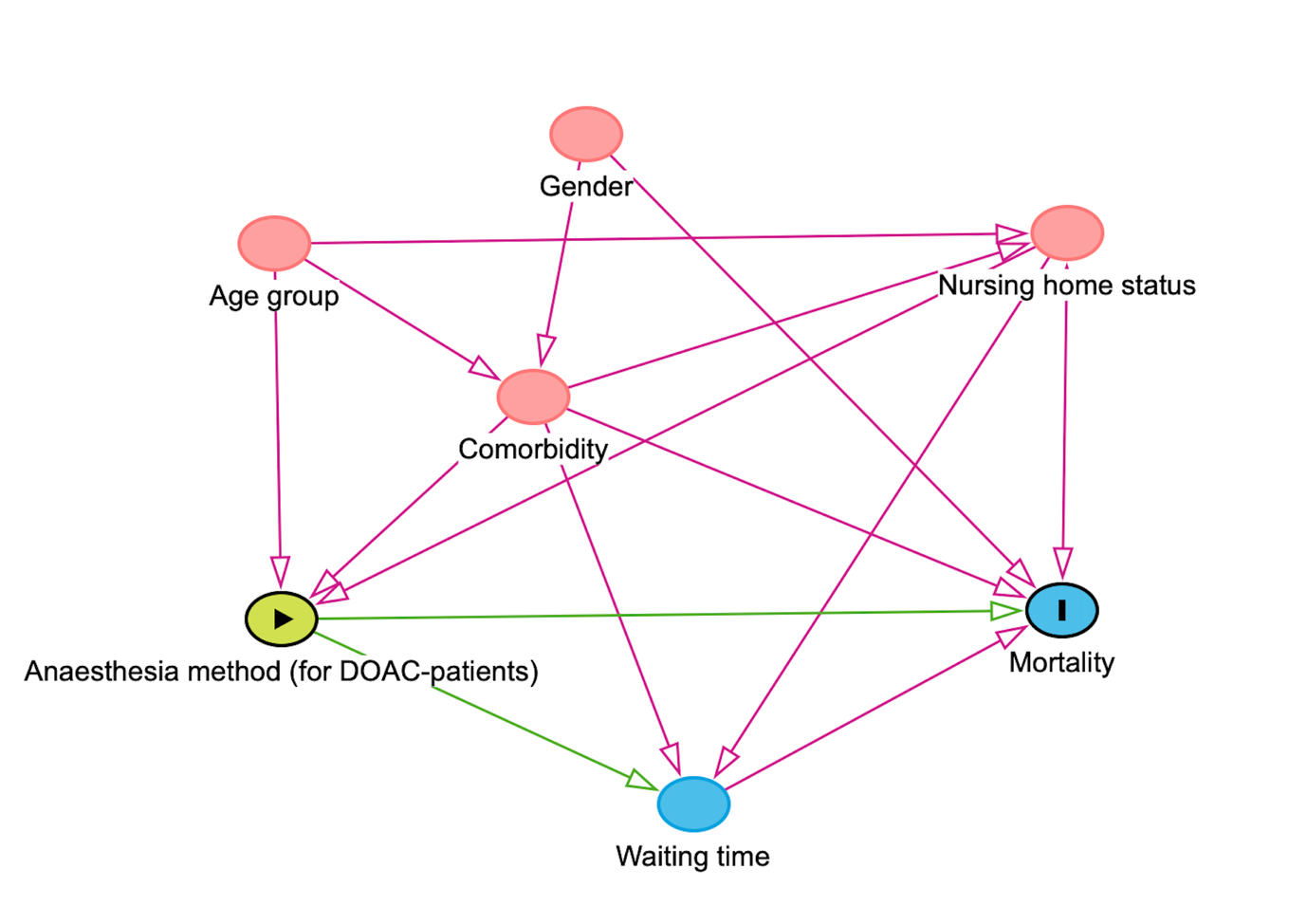


Appendix figure 2: Directed Acyclic Graph for the adjustment model for the association between DOAC and the outcomes waiting time or length of stay. The choice of anesthesia is a mediator between DOAC use and the outcomes, and should not be adjusted for, while nursing home status is not affecting DOAC use, and is therefore not a confounder and should not be adjusted for.


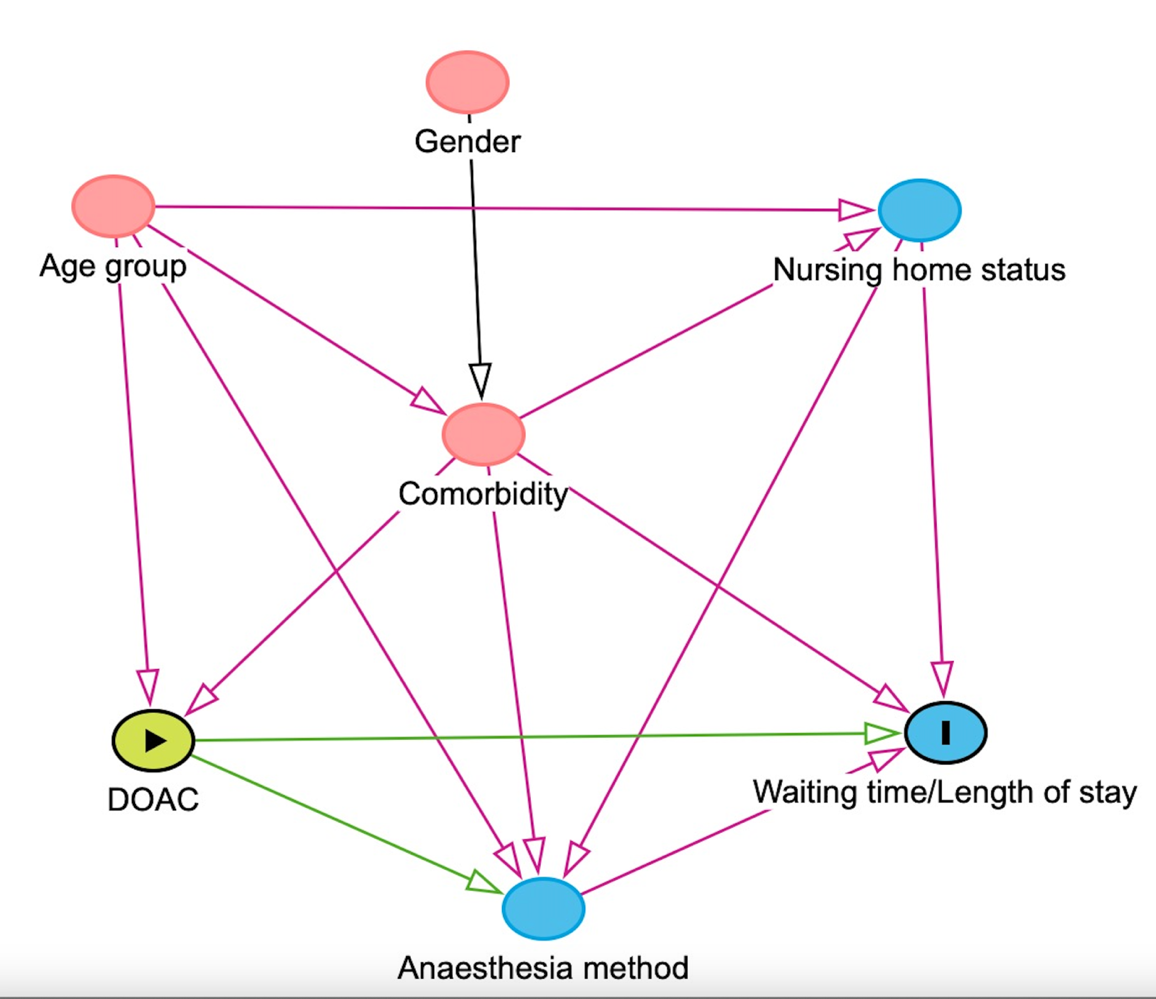

Supplement: Supplementary file 1 — Supplementary file1 (DOCX 9775 KB) [file 68_2024_2532_MOESM1_ESM.docx]
